# The Name of *Cannabis*: A Short Guide for Nonbotanists

**DOI:** 10.1089/can.2016.0027

**Published:** 2016-10-01

**Authors:** Antonino Pollio

**Affiliations:** Dipartimento di Biologia, Università Federico II, Napoli, Italia.

**Keywords:** cannabis nomenclature, cultivar, indica, sativa, species, strain

## Abstract

The genus *Cannabis* (Family Cannabaceae) is probably indigenous to wet habitats of Asiatic continent. The long coexistence between mankind and *Cannabis* led to an early domestication of the plant, which soon showed an amazing spectrum of possible utilizations, as a source of textile fibers, as well as narcotic and psychoactive compounds. Nowadays, the specie(s) belonging to the genus *Cannabis* are represented by myriads of cultivated varieties, often with unstable taxonomic foundations. The nomenclature of *Cannabis* has been the object of numerous nomenclatural treatments. Linnaeus in *Species Plantarum* (1753) described a single species of hemp, *Cannabis sativa*, whereas Lamarck (1785) proposed two species of *Cannabis*: *C. sativa*, the species largely cultivated in Western Continent, and *Cannabis indica*, a wild species growing in India and neighboring countries. The dilemma about the existence of the species *C. indica* considered distinct from *C. sativa* continues up to present days. Due to their prevalent economic interest, the nomenclatural treatment is particularly important as far as it concerns the cultivated varieties of *Cannabis*. In this context, we propose to avoid the distinction between sativa and indica, suggesting a bimodal approach: when a cultivar has been correctly established. It could be advisable to apply a nomenclature system based on the International Code of Nomenclature for Cultivated Plants (ICNCP): it is not necessary to use the species epithets, *sativa* or *indica*, and a combination of the genus name and a cultivar epithet in any language and bounded by single quotation marks define an exclusive name for each *Cannabis* cultivar. In contrast, *Cannabis* varieties named with vernacular names by medical patients and recreational users, and lacking an adequate description as required by ICNCP, should be named as *Cannabis* strain, followed by their popularized name and without single quotation marks, having in mind that their names have no taxonomical validity.

## Introduction

Depending on the taxonomical treatment adopted,^1^ the genus *Cannabis* (Hemp, Family Cannabaceae) includes up to three species, each with a very long history of domestication. Plants belonging to this genus are probably indigenous to the Asiatic Continent, where they preferably grew in wet places and near water bodies.^2^ This kind of environment was also frequently chosen as a temporary settlement by human nomadic groups, before the discovery and diffusion of agricultural techniques.^3^ *Cannabis* species in the wild had a weedy attitude, growing in soils with high concentrations of nitrogen released by animal dejections and human activities.^2^ The long coexistence between mankind and hemp led to an early domestication of the plant, which soon showed an amazing spectrum of possible utilizations. Hemp has been used as a source of textiles, as an edible plant,^4^ and as a medicinal and psychoactive plant^5^ (resins produced by secretory glandular trichomes). In recent times, hemp fibers have been used to produce bioplastic and antibacterial agents; moreover, the trichomes are considered as biofactories of phytochemicals with multiple biotechnological applications.^6^ The extent of *Cannabis* domestication has been so persistent to cause the disappearing of the wild species: nowadays, the specie(s) belonging to the genus *Cannabis* are represented by myriads of cultivated varieties, which occasionally escape cultivation and grow also in the wild, giving life to forms that lose some features typical of cultivated ones. For this reason, the nomenclature of *Cannabis* has unstable foundations and has been the object of numerous taxonomic treatments. To fully understand the difficulties in applying a shared nomenclature to *Cannabis*, a digression is necessary to describe what is a species (Table 1) and what means to give a name to a species.

**Table 1.** **What Is a Species: A Biological (and Nomenclatural) Dilemma**

“There is no consensus on how to define a species, and likely never will be.”^19^ Despite this discouraging preamble, we will try to present some basic information. The following definitions are among the most diffused in the species-definition debate over the last 50 years.
Biological species concept^30^
“Species are groups of interbreeding natural populations that are reproductively isolated from other such groups.”
Diagnostic concept ^31^
A species can be defined as “the smallest aggregation of population (sexual) or lineages (asexual) diagnosable by a unique combination of character states in comparable individuals.”^31^ According to this definition, a species is limited by a definite set of characters, which, traditionally, are morphological.
Genealogical species concept^32^
A species is represented by populations that constitute a single group, without any exclusive subgroup. All the members of the group share a common ancestor (monophyly).
Ecological species concept^33^
“A species is a lineage (or a closely related set of lineages), which occupies an adaptive zone minimally different from that of any other lineage in its range and which evolves separately from all lineages outside its range. A lineage is a clone or an ancestral-descendent sequence.”
There are unsolved difficulties in applying any of the definitions listed above, whatever the adopted: “every group differs in the biological criteria impacting species divergence, setting up a sliding scale from well defined to problematic species.”^34^

## An Outline of Nomenclatural Rules in Botany

Naturals sciences rely on shared nomenclatural rules. Although this statement sounds obvious now, it was not so for centuries, until at the beginning of the 18th Century became evident that there was a need to develop efficient nomenclatural tools for handling an increasing number of organisms. Naturalists during the 16th and 17th Centuries applied to species names that were actually short descriptions (polynomial system). The tendency toward a simplification of nomenclature was already evident in the *Pinax Theatri Botanici*, written in 1623 by Caspar Bauhin,^7^ but only in the mid of 18th century Carl von Linnaeus provided a new framework to nomenclature, recommending in his *Species Plantarum*^8^ that each species should be designated by a *nomen trivialis*, formed by the union of the generic name with a single word (epithet). By the second half of 18th century, this binomial nomenclature was adopted worldwide, and the need for a set of nomenclatural rules was already raised by JB Lamarck at the end of the same Century.^9^ The first formalized laws for the nomenclature of plant species were prepared by Alphonse De Candolle in 1867,^10^ but only in the 20th century both botanists and zoologists produced Codes of nomenclature, accepted by the international community of scholars. As far as botany is concerned, the International Code of Botanical Nomenclature has set up the rules for naming plants starting from the International Botany Congress held in Vienna in 1905.^11^ However, the first Code accepted by the botanist community is the Cambridge International Code^12^ and only after the Second World War a regular update of the Code has been carried out every 6 years. Since its last edition,^13^ the Code has been renamed as the International Code of Nomenclature for Algae, Fungi, and Plants (ICN). The system proposed by the ICN is closed and hierarchically arranged (Table 2).

**Table 2.** **A Simplified Summary of the Hierarchical Organization of the International Code of Nomenclature for Algae, Fungi, and Plants**

Taxon: a taxonomic group of any rank
The taxa of one rank exclude each other
The name of a taxon is ruled by:
1. Publication validity;
2. Priority;
3. Typification;
The species is the core taxon of the system
The rank below the species is the *varietas*
*Varietates* showing pattern of affinity are grouped into *subspecies*

A taxonomic group of any rank (generically called taxon) can be considered as valid if: (1) it has been regularly published; (2) it has not been diversely and correctly named before (priority); and (3) it has been typified. A type is a material on which the description of a taxon is based. In the case of a plant species, it is generally a herbal specimen. The specimen on which the description is based is called the *holotype* (Table 3).

**Table 3.** **Handling Nomenclature Principles**

How to give a valid name to a species—some basic rules
1. Check if your putative new species has been already described and correctly erected. If not:
2. Write a protologue, which is a description of the morphological diagnostic features of the new species, and draw a sketch of the specimen (the *iconotypus*). Description and drawings should be carried out on an individual plant that represents the species: the *holotypus.*
3. The *holotypus* should be preserved in an official repository (i.e., an Herbarium).
When a name of a species need to be reexamined
1. If it is a *nomen nudum* (someone gave a name, but he didn't write the protologue)
2. If the same species has already been correctly named (priority)
3. If it has not been typified

All nomenclatural rules included in the Code are based on the taxon system, and this architecture raises some important questions. The hierarchical system of taxa, although the Code is scientifically neutral and provides only a series of conventional rules, is deeply rooted into evolutionary theory. Most practitioners in nomenclature consider a taxon as a monophyletic entity^14^ and arrange the nomenclature according to the current opinions on plant phylogeny. This tendency is particularly evident when new taxa are created or separated following molecular approaches, for example, DNA barcoding.^15^ What is the position of cultivated plants like *Cannabis* in this framework? It is acknowledged that the botanical entities known as cultivated varieties are a product of human selection and cannot be assimilated to wild *varietates*. In contrast to these latter, cultivated varieties (cultivars) are a product of human activity and are not subjected to the selective pressure of the environment.^16^ This argument has been largely debated, and the idea that cultivars should be considered as a separate matter is not new. Linnaeus was the first to place cultivated plants under a separated category, suggesting the adoption of different nomenclatural rules for them.^17^ However, it was not until 1953 that the first edition of the International Code of Nomenclature for Cultivated Plants (ICNCP) was published.^18^

The first and foremost principle of the ICNCP is that the names of cultivated plants cannot be handled using the system of taxon, which is replaced by the culton (a systematic group of cultivated plants).^19^ The core entity of the nomenclatural system for cultivated plants is the cultivated variety or cultivar (Table 4). Each cultivar is the product of human selection and is directed toward definite goals related to human activities. The cultivar can be reproduced and is not subjected to extinction. The nomenclatural system of cultivars is open: each name of a cultivar is not exclusive and the same cultivar could have different names, depending on the scope of the classification.^16^ Cultivars are static units; they are defined by a set of characters and are linked to a standard, generally a specimen, or a document.^19^

**Table 4.** **Some Basic Rules for the Nomenclature of Cultivated Plants**

Culton: a systematic group of cultivated plants
Cultivar: a cultivated variety, uniform and stable in its characters
Group: an assemblage of similar cultivars on the basis of defined characters
The name of a cultivar or Group is the combination of the genus, or lower taxon to which it is assigned, with a cultivar or group epithet
The epithet can be a vernacular word of any language and should be not written in italics
The epithet is bounded by single quotation marks

## The Classification of *Cannabis*

The existence of cultivated and wild entities of hemp dates back to Dioscorides and passing from the physicians and botanists of the Renaissance (the German botanist Leonardt Fuchs was the first to adopt the term sativa, for indicating the domesticated hemp^20^) survived until the 18th Century, when Linnaeus in *Species Plantarum*^8^ described a single species of hemp, *Cannabis sativa*. Later, Jean-Baptiste Lamarck^9^ proposed two species of *Cannabis*: *C. sativa*, the species largely cultivated in the western continents, and *Cannabis indica*, a wild species growing in India.^21^ The taxonomic treatment of Lamarck was rejected about 50 years later by J. Lindley,^22^ who restricted *Cannabis* to *C. sativa*, following Linnaeus' classification, and the concept of *Cannabis* as a monospecific genus was confirmed in the following century. Only in the second decade of 1900's a new species, *Cannabis ruderalis*,^23^ was erected, whereas the reinstatement of the species *C. indica* was more recently suggested by Schultes et al.^24^ In more recent times, genomic DNA studies to classify *C. sativa* have been carried out using *Cannabis* varieties of different geographical origin. The results seem to suggest that a polytypic concept of *Cannabis* cannot be ruled out.^25^ In addition, chemotaxonomical markers are a promising tool to identify different *Cannabis* accessions and to screen hybrids, taking into account that all *Cannabis* varieties intercross successfully and produce fertile hybrids.^26^

A biphasic approach, combining morphological and chemical characters (fruit morphology and Δ^9^-tetrahydrocannabinol [THC] content) was adopted by Small and Cronquist,^1^ who recognized the following four *Cannabis* taxa (all belonging to the single species *C. sativa*) that “coexist dynamically by means of natural and artificial selection”:

1. *Cannabis sativa* L. subsp. *sativa* var. *sativa*;
2. *Cannabis sativa* L. subsp. *sativa* var. *spontanea* Vavilov;
3. *Cannabis sativa* L. subsp. *indica* Small & Cronquist var. *indica* (Lam) Wehmer;
4. *Cannabis sativa* L. subsp. *indica* Small & Cronquist var. *kafiristanica* (Vavilov) Small & Cronquist.

According to the authors, both *varietates* belonging to the subspecies sativa are common in North America, Europe, and Asia and show a limited intoxicant potential. In contrast, the *varietates* of the subspecies indica have high intoxicant potential and grow mainly in the Asiatic Continent.

Recently, Small^2^ has proposed two possible classification of *Cannabis*, one based on ICP, which confirms his previous taxonomical treatment, and a new classification system for domesticated *Cannabis*, which is based on ICNCP and recognizes six groups of cultivars as follows:

1. Group of the non-narcotic plants, domesticated for stem fiber and/or oil seed in Western Asia and Europe. Low THC and high cannabidiol (CBD);
2. Group of the non-narcotic plants domesticated in East Asia, mainly China. Low to moderate THC, high CBD;
3. Group of the narcotic plants domesticated in South Central Asia. High cannabinoids, mostly THC;
4. Group of the narcotic plants domesticated in South Asia (Afghanistan and neighboring Countries), contains both THC and CBD.

In addition, there are also at least two stabilized hybrid groups with intermediate characters between the four groups (Table 5).

**Table 5.** **Floral Characteristics of *Cannabis***

In 95% of Angiosperms (flowering plants), the flower contains both male and female reproductive structures, but in the remaining 5%, flowers bear either male or female reproductive structures. If the same individual bears both male and female flowers the plant is called monoecious, and if male and female flowers are produced by different individuals the plant is called dioecious.
*Cannabis* is a genus characterized by dioecy, with male individuals showing short life cycle, and higher and slimmer shoots compared to female ones, but cultivars that produce also hermaphrodite or monoecious flowers (bearing separate male and female flowers on the same individual) are well known.^35^
Hybridization is the merging of differing gene pools to create offspring. *Cannabis* is wind pollinated; male plants produce vast amounts of pollen that can spread over large geographical areas, allowing the pollination of female flowers of plants growing very far from pollen-bearing flowers.
The extensive cultivation of *Cannabis* plants and the absence of barriers, which reduce or constrain interbreeding, lead to the production of numerous fertile hybrids that can maintain their characteristics over different generations.^1,24^

This recent systematic treatment calls attention to the still existing practical difficulties of applying the International Code of Nomenclature to the genus *Cannabis*. Small^2^ is careful in the application of the code, and this cautious attitude is the consequence of the perplexity about considering *Cannabis* exclusively as a cultivated plant. The studies of last two decades suggest that *Cannabis*, as other crops, exists in the so called crop–weed complexes, which are formed by cultivated forms and weedy forms escaped from cultivations and growing in the wild. These latter can establish new characters and are newly under the pressures of natural selection. Thus, it seems difficult to circumscribe *Cannabis* solely as a cultivated plant. In our opinion, an application of the taxon system to the genus *Cannabis* together with the *sativa*/*indica* distinction should be avoided, as recently suggested.^28^ Due to the prevalent economic interest of the cultivated varieties of *Cannabis*, a simplified nomenclature system based on ICNCP should be applied. According to ICNCP, it is not mandatory to use the species epithets, *sativa* or *indica*, and a combination of the genus name and a cultivar epithet, in any language and bounded by single quotation marks (i.e., *Cannabis* ‘fibranova’, to cite a cultivar largely cultivated for fiber production), defines an exclusive name for each *Cannabis* cultivar.

However, due to its numerous medical and recreational usages, hundreds of *Cannabis* cultivated varieties have been developed and named with vernacular names by medical patients and recreational users. Few of these can be treated as real *Cannabis* cultivars, having been regularly named and registered according to the ICNCP, but many others, particularly marijuana strains, lack an adequate description and a standard. For this reason, their names cannot be accepted as cultivar epithets. Any strain that has not been formally described as a cultivar, for example, the so called Sour diesel, or Granddaddy Purple, should be named as follows: *Cannabis* strain Sour diesel, or strain Granddaddy Purple, with their popularized name without single quotation marks, having in mind that their names have no taxonomical validity.

## Abbreviations Used

CBD: cannabidiol
ICNCP: International Code of Nomenclature for Cultivated Plants
ICN: International Code of Nomenclature for Algae, Fungi, and Plants
THC: tetrahydrocannabinol
